# The role of glutamine metabolism in the pathogenesis of idiopathic pulmonary fibrosis and its therapeutic potential

**DOI:** 10.3389/fonc.2026.1873614

**Published:** 2026-09-01

**Authors:** Junzi Li

**Affiliations:** Department of Respiratory and Critical Care Medicine, Henan Province Hospital of Traditional Chinese Medicine, The Second Affiliated Hospital of Henan University of Traditional Chinese Medicine, Zhengzhou, China

**Keywords:** glutamine metabolism, glutaminolysis, idiopathic pulmonary fibrosis, immunometabolism, mTOR/AMPK, myofibroblast

## Abstract

Idiopathic pulmonary fibrosis (IPF) is a progressive, fatal interstitial lung disease characterized by usual interstitial pneumonia, relentless decline in lung function and incomplete disease modification by available antifibrotic therapies. Beyond canonical profibrotic pathways, accumulating evidence identifies aberrant glutamine metabolism as a convergent metabolic feature of IPF pathogenesis. In structural cells, glutaminolysis fuels myofibroblast activation, *de novo* glycine and proline synthesis for collagen, whereas epithelial glutamine utilization may support antioxidant defence, mitochondrial adaptation and repair. In immune cells, glutamine shapes macrophage and T-cell polarization and may contribute to an inflammatory, profibrotic microenvironment. Glutamine-derived intermediates intersect with mTOR/AMPK and TGF-*β*/Smad signalling and act as cofactors for epigenetic regulators, thereby stabilizing apoptosis-resistant, profibrotic transcriptional programmes. Experimental models and human studies further demonstrate upregulation of glutamine transporters and glutaminase 1 (GLS1) in fibrotic lungs, protection from bleomycin-induced fibrosis after genetic or pharmacologic GLS1 inhibition, and a genetic association between lower circulating glutamine and increased IPF risk. Importantly, the effects of glutamine metabolism are context- and cell type–dependent: epithelial and immune glutamine utilization may support repair, barrier integrity and host defence, whereas excessive fibroblast-directed glutaminolysis promotes matrix accumulation. Building on these observations, this review synthesizes current knowledge on cell type–relevant glutamine metabolism in lung fibrosis, distinguishes direct lung-fibrosis evidence from extrapolated mechanistic evidence, delineates its integration with fibrogenic signalling, oxidative stress, mitochondrial stress and immunometabolism, and critically evaluates the therapeutic potential and caveats of targeting glutamine uptake, catabolism and nutrient-sensing pathways as adjuncts to existing antifibrotic regimens.

## 1 Introduction

Idiopathic pulmonary fibrosis (IPF) is a chronic, progressive, and ultimately fatal fibrosing interstitial lung disease (ILD) of unknown etiology, predominantly affecting elderly individuals and carrying one of the most dismal prognoses among all respiratory disorders. According to the joint clinical practice guidelines endorsed by the American Thoracic Society (ATS), European Respiratory Society (ERS), Japanese Respiratory Society (JRS), and Asociacion´ Latinoamericana de Torax´ (ALAT), IPF is defined by the histopathological and radiological pattern of usual interstitial pneumonia (UIP), characterized by spatial and temporal heterogeneity, honeycombing, and the presence of fibroblastic foci (1). The clinical course is relentlessly progressive, manifesting as worsening exertional dyspnea, persistent dry cough, and inexorable pulmonary function decline culminating in respiratory failure (2). The underlying pathogenesis involves repetitive micro-injuries to the alveolar epithelium that trigger a dysregulated wound-healing cascade, resulting in aberrant activation of fibroblasts and myofibroblasts, excessive extracellular matrix (ECM) deposition, and irreversible destruction of lung parenchymal architecture (3).

Epidemiologically, IPF represents a significant and growing global health burden. A landmark systematic review documented an increasing temporal trend in IPF incidence, with annual rates in Europe and North America ranging from 3 to 9 cases per 100,000 (4). A subsequent multi-country analysis reported adjusted global incidence estimates of 0.09–1.30 per 10,000 persons and prevalence estimates of 0.33–4.51 per 10,000, with North America consistently demonstrating the highest regional figures (5). A broader systematic review encompassing diverse ILD subtypes confirmed substantial variability in reported IPF prevalence—ranging from 7 to 1,650 per 100,000—attributable largely to heterogeneity in diagnostic criteria and data capture methodologies (6). Most recently, a large-scale meta-analysis incorporating 26 studies estimated a pooled global incidence of 5.8 per 100,000 per year and pooled global prevalence of 17.7 per 100,000, with notably elevated estimates in North America (incidence 9.0; prevalence 27.2 per 100,000) relative to Europe and Asia (7). Irrespective of regional variation, the overarching trajectory reflects a rising disease burden, compounded by a median post-diagnosis survival of only 2–5 years and substantial disease-related mortality.

Establishing an accurate diagnosis of IPF requires systematic exclusion of other identifiable causes of ILD and relies primarily on HRCT identification of a characteristic UIP pattern, supported by histopathological confirmation via surgical lung biopsy or transbronchial cryobiopsy in appropriate clinical contexts (8, 9). Despite refinements in imaging technology and multidisciplinary assessment protocols, early-stage diagnosis remains challenging, and many patients present at an advanced phase of disease. The considerable clinical heterogeneity of IPF—encompassing variable rates of progression, divergent comorbidity profiles, and distinct molecular endotypes—has stimulated growing interest in precision medicine approaches, including genomic classifier testing and the identification of validated theragnostic biomarkers; however, these strategies remain largely investigational and have yet to be integrated into routine clinical practice (10).

The pharmacological management of IPF has historically been anchored by the antifibrotic agents pirfenidone and nintedanib, both of which attenuate the annual rate of forced vital capacity (FVC) decline and reduce the risk of acute exacerbations (11). More recently, nerandomilast, an oral preferential phosphodiesterase 4B inhibitor, has further expanded the therapeutic landscape after phase III evidence showed a smaller decline in FVC over 52 weeks compared with placebo, including in patients receiving background antifibrotic therapy (12). Nevertheless, available therapies still do not reverse established fibrosis, restore destroyed alveolar architecture or fully overcome tolerability and adherence limitations. An expanding therapeutic pipeline encompasses novel mechanistic targets—including additional phosphodiesterase 4 inhibitors, lysophosphatidic acid receptor antagonists, *α*v*β*6/*α*v*β*1 integrin inhibitors and the prostacyclin agonist treprostinil—several of which have demonstrated promising signals in phase II trials (13). However, phase III outcomes have remained mixed; for example, the ZEPHYRUS-1 randomized clinical trial evaluating pamrevlumab, a monoclonal antibody targeting connective tissue growth factor activity, failed to achieve a statistically significant reduction in FVC decline compared with placebo (14). Beyond disease modification, the symptomatic burden of IPF—particularly refractory chronic cough—represents a distinct unmet need; the PACIFY COUGH trial recently demonstrated that low-dose controlled-release morphine significantly reduced objective cough frequency in IPF patients (15). Collectively, these findings underscore the persistent need for mechanistically novel therapeutic approaches that complement existing antifibrotic strategies.

An evolving paradigm in IPF research has increasingly implicated aberrant cellular metabolism as a fundamental dimension of disease pathogenesis. Among the metabolic alterations now recognized in fibrotic lung remodeling, dysregulation of glutamine metabolism—or glutaminolysis—has emerged as a particularly compelling axis. Glutaminolysis involves the enzymatic cleavage of glutamine to glutamate by glutaminase (GLS), followed by its conversion to *α*-ketoglutarate (*α*-KG), which replenishes the tricarboxylic acid (TCA) cycle, supports key biosynthetic processes, and activates fibrogenic signaling nodes including mTOR complex 1 (16). Landmark experimental studies demonstrated that human lung myofibroblasts undergo glutaminolytic reprogramming mediated by upregulation of GLS1; fibroblastspecific genetic ablation of GLS1 conferred significant protection against bleomycin-induced pulmonary fibrosis in mice, and pharmacological inhibition with the GLS1 inhibitor CB-839 attenuated fibrosis in both bleomycin- and TGF-*β*1–driven experimental models, providing strong preclinical rationale for targeting this pathway therapeutically (17). More broadly, metabolic reprogramming across multiple IPF-relevant cell populations—including myofibroblasts, alveolar epithelial cells, macrophages and endothelial cells—has been shown to drive profibrotic mechanisms encompassing autophagy impairment, cellular senescence, oxidative stress amplification and dysregulated inflammatory responses (18).

However, how dysregulated glutamine metabolism integrates with core fibrogenic pathways and how tractable this axis is as a therapeutic target in IPF remain incompletely defined. This review therefore summarises evidence from IPF, experimental pulmonary fibrosis and selected mechanistic models, critically distinguishes direct lung-fibrosis findings from extrapolated evidence, and highlights key mechanistic and translational questions for future research.

## 2 Overview of glutamine metabolism and its regulation in lung cells

Glutamine is the most abundant free amino acid in plasma and intracellular fluids and becomes conditionally essential in situations of rapid proliferation, stress, or tissue injury. In the lung, which is continuously exposed to high oxygen tension, mechanical stretch, and environmental noxae, glutamine metabolism supports bioenergetic needs, macromolecular synthesis, and redox homeostasis in several structural and immune cell populations. In this section, we briefly summarize the main biochemical routes of glutamine metabolism, the principal regulatory pathways controlling glutamine utilization, and the cell type–specific features that are most relevant to fibrotic lung remodeling.

### 2.1 Biochemical pathways of glutamine metabolism

After transport into the cell through dedicated or functionally coupled amino-acid transport systems, glutamine enters a network of reactions that can be schematically divided into three main branches: deamidation and anaplerosis into the tricarboxylic acid (TCA) cycle, nitrogen donation to nucleotide and non-essential amino acid synthesis, and support of redox balance through NADPH and glutathione (GSH) metabolism. SLC1A5/ASCT2 is a major sodium-dependent glutamine transporter, whereas SLC7A5/LAT1 mainly functions as a sodium-independent exchanger for large neutral amino acids and uses intracellular glutamine as an exchange substrate to support uptake of essential amino acids such as leucine, thereby linking glutamine availability to mTORC1 activation. Additional transporters, including SLC38A2/SNAT2, may also participate in glutamine- and alanine-linked metabolic reprogramming in fibrotic contexts, as discussed below.

The first committed step of glutamine catabolism is the hydrolysis of glutamine to glutamate and ammonia catalyzed by glutaminase (GLS). Mammalian cells express two glutaminase genes, GLS (GLS1, kidneytype) and GLS2 (liver-type), each producing several splice isoforms with distinct tissue distribution and regulatory properties (19–23). These enzymes are predominantly localized in mitochondria, which places glutamine deamidation in direct continuity with mitochondrial metabolism. Glutamate is then converted to *α*-ketoglutarate (*α*-KG) either by glutamate dehydrogenase or by various aminotransferases. The resulting *α*-KG enters the TCA cycle and provides anaplerotic carbon to sustain oxidative metabolism and to replenish intermediates that are continuously withdrawn for biosynthetic purposes (24).

Beyond this classical glutaminolysis, an alternative glutaminase II pathway has been described in rapidly proliferating cells. In this pathway, a glutamine transaminase converts glutamine and a suitable *α*-keto acid into *α*-ketoglutaramate, which is subsequently hydrolyzed by *ω*-amidase to form *α*-KG and ammonia. This route can provide anaplerotic carbon independently of GLS1/GLS2 activity and remains active when classical glutaminase is pharmacologically inhibited, as shown in tumour models (22). These data emphasize that glutamine-derived anaplerosis is supported by more than one enzymatic system and that complete suppression of glutamine entry into the TCA cycle may require the combined targeting of several enzymes.

Glutamine is also a major nitrogen donor. Its amide nitrogen is used by multiple glutamine-dependent amidotransferases involved in *de novo* purine and pyrimidine synthesis, glucosamine biosynthesis, and other nitrogen transfer reactions, whereas the *α*-amino nitrogen is retained in glutamate and subsequently enters aminotransferase reactions after glutamine-to-glutamate conversion. Tracer studies in transformed cells have shown that glutamine consumption often exceeds the minimum requirement for protein and nucleotide synthesis; excess glutamine carbon is oxidized in the TCA cycle and channelled into lipid and non-essential amino acid synthesis, whereas nitrogen is largely released as alanine and ammonia (24). Glutamate, generated from glutamine, is in addition a direct precursor for several non-essential amino acids and a key substrate for GSH synthesis. Limiting glutamine availability or inhibiting GLS reduces GSH levels and compromises antioxidant defences (19, 25). In experimental systems, enhanced glutamine metabolism can provide reductive power and nucleotides required for DNA repair, thereby contributing to radioresistance, whereas combined inhibition of the pentose phosphate pathway and glutaminase sensitizes cells to irradiation (26).

Taken together, these studies indicate that glutamine metabolism contributes simultaneously to TCA anaplerosis, biosynthetic nitrogen supply, and maintenance of redox homeostasis. In the lung, where cells are exposed to oxidative stress and recurrent damage–repair cycles, such functions are likely to be particularly important for preserving epithelial integrity and cell viability.

### 2.2 Regulatory networks controlling glutamine utilization

Glutamine metabolism is subject to tight regulation by nutrient-sensing and stress-responsive pathways. Among these, the mechanistic target of rapamycin complex 1 (mTORC1), c-Myc, hypoxia-inducible factor-1*α* (HIF-1*α*), AMP-activated protein kinase (AMPK), and the autophagy–mitochondria axis are especially relevant.

mTORC1 integrates signals from amino acids, growth factors, and energy status to coordinate cell growth and metabolism. It stimulates glutamine catabolism by repressing the mitochondrial deacylase SIRT4, thereby relieving its inhibitory effect on glutamate dehydrogenase and favouring the conversion of glutamate to *α*-KG (27). Amino acids activate mTORC1 through both Rag-dependent and Rag-independent mechanisms. Leucine and arginine signal via Rag GTPases, whereas glutamine and asparagine can activate mTORC1 through an ADP-ribosylation factor 1–dependent pathway that does not require the Rag GTPases but still depends on lysosomal localization (28). In parallel, glutaminolysis-derived *α*-KG contributes to mTORC1 activation, while glutamine deprivation or inhibition of GLS leads to AMPK activation, mTORC1 inhibition, and induction of autophagy (29). Recent work has shown that glutamine can regulate the AMPK– mTORC1 axis not only via classical glutaminolysis but also through an asparagine synthetase–GABA shunt branch, indicating that multiple glutamine-dependent circuits converge on mTOR signalling and macroautophagy.

c-Myc is a central transcriptional regulator of glutamine metabolism. It upregulates GLS1 and glutamine transporters and promotes the glutamine-addicted phenotype observed in various tumour cells. In MYCdriven multiple myeloma, GLS1 is required to sustain mitochondrial function and proliferation, and pharmacological inhibition of GLS1 selectively affects MYC-overexpressing cells (30). These observations further support the broader MYC-linked glutamine dependency described in the same study (30). At the same time, the inhibition of glutaminase can be compensated for by increased activity of amidotransferases and by enhanced glycolysis, so that combined inhibition of several enzymes is needed to fully block glutamine catabolism (31). These findings illustrate the metabolic flexibility of glutamine pathways, which is likely to be relevant also in chronically injured lung tissue.

HIF-1 *α* is stabilized under hypoxic conditions and orchestrates a broad metabolic reprogramming that includes a shift from oxidative phosphorylation to glycolysis, increased glycogen synthesis, and a tendency to use glutamine rather than glucose as a carbon source for fatty acid synthesis (32). HIF-1 can also be activated by oncogenic PI3K/Akt/mTOR signalling, thereby linking growth factor signals to glutaminedependent adaptations (33). In fibrotic lungs, characterized by regional hypoxia and microvascular alterations, similar mechanisms may contribute to the preferential use of glutamine by fibroblasts and endothelial cells.

AMPK acts as an energy sensor activated by an increased AMP/ATP ratio or upstream kinases. Under low-glucose conditions, AMPK phosphorylates the scaffold protein PDZD8, which enhances its interaction with GLS1 and promotes glutaminolysis, thereby shifting carbon utilization from glucose to glutamine and sustaining TCA flux in skeletal muscle and macrophages (34). This AMPK–PDZD8–GLS1 axis exemplifies how glutamine can serve as an alternative fuel during energy stress. In parallel, AMPK and mTORC1 jointly regulate autophagy, which in turn contributes to the provision of amino acids, including glutamine, through lysosomal degradation. Autophagy has been implicated in the control of glucose, lipid, and glutamine metabolism as well as in cell survival and chemoresistance in cancer and immune cells (35). Although data in IPF are still limited, the convergence of altered autophagy, mitochondrial dysfunction, and enhanced glutaminolysis in several lung cell populations suggests that this regulatory layer is also relevant in the fibrotic setting.

### 2.3 Cell type–relevant features of glutamine metabolism in the lung

Direct evidence for cell type specificity remains strongest for AT2 cells and fibroblasts, whereas information on pulmonary endothelial and immune cells is more limited and is partly extrapolated from broader cell biology. Therefore, this section summarizes cell type–relevant observations rather than assigning unique glutamine pathways to each lung cell population.

Alveolar type II (AT2) cells act as progenitors for the alveolar epithelium and are responsible for surfactant production and epithelial regeneration. Single-cell transcriptomic and metabolomic analyses in IPF and bleomycin-induced fibrosis models showed a downregulation of glutamine catabolic enzymes in AT2 cells, suggesting impaired glutamine utilization with intracellular accumulation (36). Inhibition of GLS1 or glutamic-pyruvate transaminase-2 in AT2 cells reduces proliferation and differentiation, indicating that intact glutamine metabolism is required for effective alveolar repair. In lung transplantation models, exogenous glutamine administration preserved alveolar structure and improved compliance and oxygenation. These effects were accompanied by mTOR activation, suppression of autophagy markers, and reduced apoptosis, and were reversed by rapamycin, supporting a role for glutamine in modulating the mTOR–autophagy axis in alveolar epithelium (37). However, because glutamine is also a standard component of most culture media, its presence in epithelial differentiation protocols should be interpreted as a requirement for general cellular homeostasis rather than definitive evidence for an AT2 lineage-specific regulatory programme.

Fibroblasts and myofibroblasts are key effector cells in pulmonary fibrosis. In fibrotic lung fibroblasts, glutaminolysis is upregulated, with increased GLS1 expression and glutamine-derived anaplerosis supporting collagen synthesis (17). Glutamine transporter-dependent metabolic dependencies have also been described in other lung disease contexts, highlighting the broader relevance of glutamine uptake pathways in lung cellular metabolism (38). In experimental pulmonary fibrosis, cathepsin K accumulates in fibroblasts and aggravates collagen deposition by promoting glutamine metabolism through an endocytosisSNX9-TGF-*β*1/SMAD3 axis, which leads to GLS1 upregulation and glutamine enrichment in MRC-5 fibroblasts and fibrotic lung models (39). Serum cathepsin K levels correlate with circulating glutamine and poor prognosis in patients, pointing to a systemic reflection of fibroblast glutamine remodelling. Caveolin-1 has been identified as a negative regulator of fibroblast glutaminolysis; its downregulation in silica-induced fibrosis is associated with YAP1 activation, GLS1 induction and increased glutaminolysis, whereas a caveolin-1–derived peptide attenuates fibroblast activation and fibrosis *in vivo* (40).

Pulmonary endothelial cells are highly glycolytic, and glutamine likely supports anaplerosis, nucleotide synthesis and redox balance as in other endothelial systems (41). However, direct evidence defining glutamine-dependent endothelial programmes in IPF lungs remains limited, and the contribution of endothelial glutamine metabolism to microvascular rarefaction or barrier dysfunction in fibrotic lungs requires further validation.

Immune cells in the lung microenvironment are also influenced by glutamine availability, but most mechanistic insights derive from non-lung or non-fibrotic settings. Macrophages and T cells use glutamine to support bioenergetic, biosynthetic and signalling demands during activation, and glutaminase-dependent metabolism can regulate T-cell differentiation and effector responses (42). In autoimmune models, glutamine blockade reduces Th1/Th17 differentiation and modulates amino-acid transporter and mTOR activity (43). These findings suggest that glutamine metabolism may shape immune-stromal cross-talk in IPF, but direct evidence in human IPF immune subsets remains incomplete.

**Figure 1:**
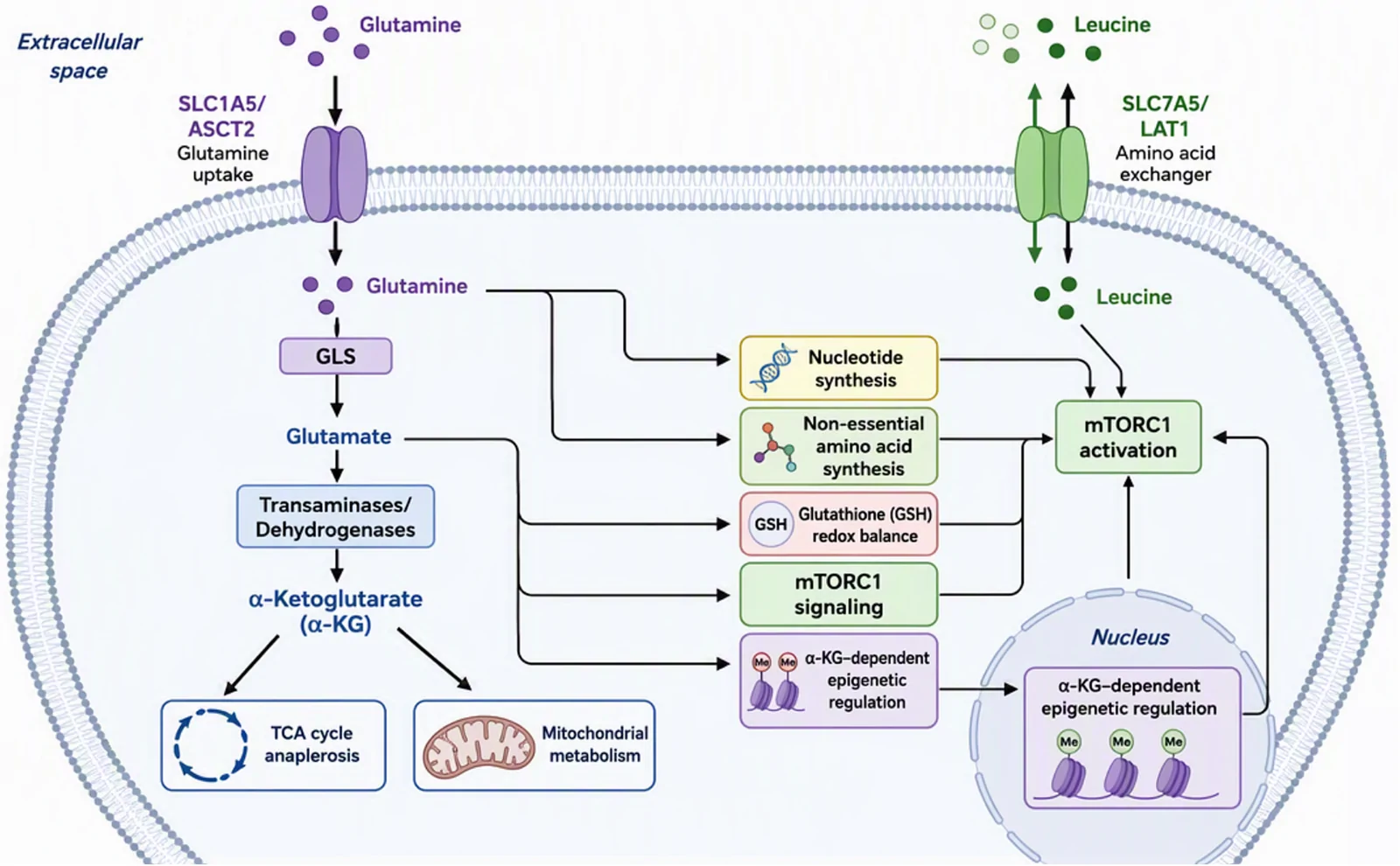
Generic cellular glutamine utilization relevant to lung fibrosis. Glutamine is imported mainly via SLC1A5/ASCT2 and exchanged through SLC7A5/LAT1, then converted by GLS to glutamate and subsequently to *α*-ketoglutarate. These metabolites support TCA cycle anaplerosis, mitochondrial metabolism, nucleotide synthesis, non-essential amino-acid synthesis, glutathione-dependent redox balance, mTORC1 signalling and *α*-KG-dependent epigenetic regulation. The schematic summarizes core mechanisms that are relevant to lung structural and immune cells, but not all pathways are directly demonstrated in every lung cell type.

In summary, current evidence supports a cell type–relevant but incompletely resolved model of glutamine metabolism in fibrotic lungs: AT2 cells appear to require glutamine-dependent pathways for repair and stress adaptation, fibroblasts exploit glutaminolysis to support collagen synthesis and profibrotic activation, and endothelial and immune-cell roles remain more largely inferred from broader metabolic studies ()?. These cellular processes are therefore presented as a generic schematic of core glutamine utilization rather than as fully defined cell type–specific pathways (**Figure 1**).

## 3 Dysregulated glutamine metabolism in the pathogenesis of IPF

Idiopathic pulmonary fibrosis develops in a lung microenvironment where structural and immune cells undergo profound metabolic reprogramming. Altered glutamine utilization is now recognized as a central feature of this reprogramming, linking energy supply, redox balance, epigenetic control and immune signalling.

### 3.1 Glutamine metabolism and myofibroblast activation

Myofibroblasts are the main source of excess ECM in IPF and require continuous carbon and nitrogen input to sustain proliferation, contractile activation and collagen synthesis. Direct evidence from lung fibroblast studies demonstrates that glutaminolysis is required for TGF-*β*1-induced myofibroblast differentiation. TGF-*β*1 increases GLS1 expression through SMAD3 and p38 MAPK activation, elevates glutamate and TCA cycle intermediates and decreases intracellular glutamine, indicating enhanced glutamine catabolism. Removal of extracellular glutamine or GLS1 silencing suppresses TGF-*β*1-induced profibrotic markers, whereas exogenous glutamate or *α*-KG restores myofibroblast activation in GLS1deficient cells (44). These findings provide direct mechanistic evidence that the glutamine–glutamate–*α*-KG axis is not merely associated with, but functionally required for, fibroblast activation.

Glutamine-derived carbon and nitrogen are also routed into collagen-relevant biosynthetic pathways. TGF-*β* promotes mitochondrial oxidation of glucose and glutamine carbons to meet the energetic demand of matrix protein synthesis and induces proline biosynthesis from glutamine in a SMAD4-dependent manner. This proline biosynthetic programme acts as a mitochondrial redox vent while simultaneously supplying substrate for matrix protein production (45).

At the level of fibroblast glutamine flux, ^13^C metabolic flux analysis demonstrates that TGF-*β*1 stimulation drives enhanced glutaminolysis in NIH-3T3 and MRC-5 fibroblasts, with [U-^13^C_5_]-glutamine carbon directed into mitochondrial metabolism and proline synthesis; tanshinone IIA suppresses GLS1 and proline-biosynthetic enzymes, reduces glutamine entry into mitochondria and proline hydroxylation, and thereby lowers collagen I/III and *α*-SMA expression (46). In human lung fibroblasts, TGF-*β*1 induces a transcriptional programme enriched in amino-acid biosynthesis, but under pyruvate-replete conditions GLS1 inhibition alone is insufficient to block collagen synthesis, because pyruvate fuels glutamate and alanine production via GDH and GPT2. Only dual targeting of GLS1 with GDH or GPT2 fully abrogates TGF-*β*1–induced fibrogenesis (47).

Data from other organs support a conserved role for glutaminolysis in fibroblast activation. In cardiac fibroblasts, TGF-*β* and scleraxis directly induce GLS1, and GLS1 blockade attenuates activation, while scleraxis loss abolishes the TGF-*β*–dependent increase in glutaminolysis (48). In cancer-associated fibroblasts, metabolic rewiring of amino-acid pathways, including glutamine-to-proline and glycine routing, supports collagen production at multiple levels (49). In hepatic stellate cells, emodin induces senescence and alleviates liver fibrosis by promoting Nur77-dependent GLS1 promoter methylation and suppressing glutaminolysis (50).

In IPF fibroblasts, reduced glutaminolysis by glutamine withdrawal or GLS1 silencing attenuates the profibrotic phenotype and broadly downregulates gene expression. The glutaminolysis derived metabolite *α*-KG serves as a cofactor for histone demethylases; reduced glutaminolysis increases H3K27me3 and its occupancy at the *Col3a1* and *Plk1* promoters, whereas *α*-KG supplementation selectively reverses some of these epigenetic changes (51). In systemic sclerosis, fibroblasts exhibit coordinated metabolic shifts, including altered glutamine pathways and tricarboxylic acid cycle intermediates, that favour profibrotic and proinflammatory signalling (52).

**Figure 2:**
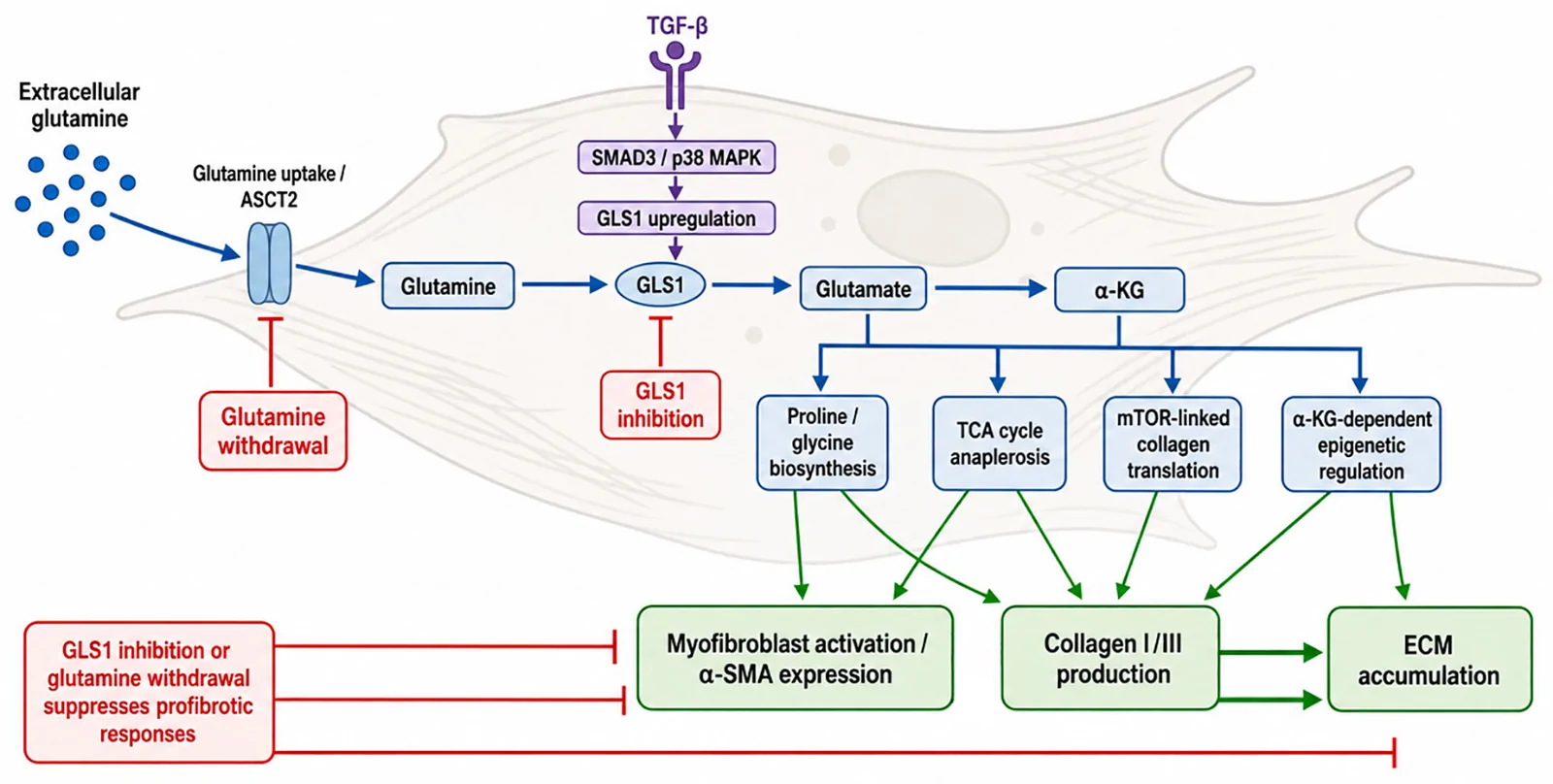
Glutamine-driven profibrotic reprogramming in lung fibroblasts during idiopathic pulmonary fibrosis. In fibrotic lung fibroblasts, extracellular glutamine is imported through glutamine transporters such as ASCT2 and converted by GLS1 to glutamate and subsequently to *α*-ketoglutarate. TGF-*β* signalling, acting through SMAD3 and p38 MAPK, promotes GLS1 upregulation and enhances glutaminolytic flux. Glutamine-derived glutamate and *α*-ketoglutarate support proline/glycine biosynthesis, TCA cycle anaplerosis, mTOR-linked collagen translation and *α*-ketoglutarate-dependent epigenetic regulation. These metabolic pathways contribute in parallel to myofibroblast activation/*α*-SMA expression and collagen I/III production, which together promote extracellular matrix accumulation. GLS1 inhibition or glutamine withdrawal suppresses these profibrotic fibroblast responses.

Taken together, these data indicate that GLS1-driven glutaminolysis, glutamate-to-*α*-KG flux, proline/glycine biosynthesis and *α*-KG-dependent epigenetic regulation are key mechanisms by which glutamine metabolism sustains collagen production and the myofibroblast phenotype in IPF and other fibrotic diseases (**Figure 2**). However, several issues limit the strength of this conclusion. Most studies rely on fibroblasts cultured in nutrient replete media with high glutamine and glucose concentrations, conditions that differ from the nutrient gradients and hypoxic niches of the fibrotic lung. In addition, fibroblasts can compensate for GLS1 inhibition by engaging alternative anaplerotic pathways, such as increased glucose oxidation or activity of amidotransferases and glutamine transaminases (22, 31, 47, 49). Future work should therefore clarify whether glutamine dependence is a stable vulnerability of IPF myofibroblasts *in vivo*, define the extent of metabolic plasticity in human disease tissue and determine whether single enzyme inhibition is sufficient or whether combinatorial targeting of several metabolic nodes will be required to achieve durable antifibrotic responses.

### 3.2 Glutamine metabolism in alveolar epithelial cell injury and repair

Alveolar epithelial injury and defective repair are central events in IPF. *In vitro*, L-glutamine is routinely included as a basal medium component in protocols used to generate AT2-like cells from murine embryonic stem cells; however, because glutamine is present in most standard culture media, this technical requirement should not be over-interpreted as direct evidence for a lineage-specific regulatory role. Rather, it indicates that adequate glutamine availability is necessary for epithelial culture homeostasis (53). More direct evidence for epithelial relevance comes from lung injury models in which glutamine supports antioxidant defence, stress adaptation and cell survival.

In primary rat AT2 cells, LPS stimulation decreases intracellular GSH, activates NF-*κ*B and increases TNF-*α* release, whereas glutamine pretreatment restores GSH levels, attenuates NF-*κ*B activation and reduces TNF-*α* secretion in a dose-dependent manner (54). Pharmacologic inhibition of GSH synthesis abolishes these effects, demonstrating that glutamine primarily modulates epithelial inflammatory responses through its role as a GSH precursor. In neonatal models of hyperoxia-induced lung injury and bronchopulmonary dysplasia, systemic glutamine administration reduces histopathological damage, oxidative stress and apoptosis, and improves lung function (55). These benefits are accompanied by downregulation of ER stress markers such as GRP78, caspase-12 and CHOP, and by suppression of stressactivated pathways including IRE1*α*/JNK and MKP-1/MAPK/cPLA2–NF-*κ*B, suggesting that glutamine mitigates hyperoxia-induced epithelial injury by limiting oxidative and ER stress (56).

Earlier studies in human A549 and rat alveolar epithelial cells showed that oxidants including cigarette smoke condensate, H_2_O_2_ and TNF-*α* provoke rapid depletion of intracellular GSH and distinct alterations in GSH-related enzymes, whereas *γ*-glutamyl transpeptidase is upregulated as part of the adaptive response, enhancing the use of extracellular GSH (57, 58). Dysregulated GSH homeostasis and oxidant/antioxidant imbalance are central features of inflammatory lung diseases, including IPF, ARDS, cystic fibrosis and asthma, with important consequences for redox-sensitive transcription factors and inflammatory gene expression (59). Taken together, these data support a protective epithelial role for glutamine as a metabolic precursor for GSH and as a contributor to stress adaptation during lung injury. In IPF, chronic oxidative stress, epithelial senescence and metabolic competition with activated stromal cells may compromise these glutamine-dependent protective mechanisms and thereby impair epithelial repair.

### 3.3 Glutamine metabolism, oxidative stress, and mitochondrial dysfunction in IPF

Oxidative stress and mitochondrial dysfunction are increasingly recognised as core components of fibrotic remodelling. Because glutamine contributes to GSH synthesis, mitochondrial anaplerosis and cellular stress adaptation, altered glutamine availability or routing can influence redox balance and mitochondrial function in IPF-relevant cell populations. To avoid repeating the epithelial redox mechanisms discussed above, this section focuses on mitochondrial and redox consequences that link glutamine metabolism to fibrotic remodelling.

In bleomycin-injured lung epithelial cells, mitochondrial respiration is severely compromised when glucose is the primary fuel but is largely preserved when glutamine is available. Glutamine supplementation increases TCA cycle intermediates and lactate while reducing DNA damage and cell death, suggesting that glutamine can substitute for glucose to sustain mitochondrial respiration and protect epithelial cells during injury (60). This finding supports the idea that epithelial glutamine utilization may be adaptive in the setting of acute or subacute lung injury, even though excessive glutaminolysis in fibroblasts may promote matrix accumulation.

In activated fibroblasts, glutaminolysis provides glutamate and *α*-KG for TCA cycle activity, biosynthesis and profibrotic signalling. Studies in systemic sclerosis fibroblasts show coordinated alterations in TCA intermediates and amino-acid metabolism, including glutamine-related pathways, in association with persistent profibrotic activation (52). In bleomycin-induced pulmonary fibrosis, tanshinone IIA reduces ROS-mediated myofibroblast activation and ECM deposition by activating Nrf2, suppressing NOX4 and redirecting glutamate toward GSH synthesis rather than TCA entry, thereby limiting glutaminolysis-driven proliferation (61). These data suggest that the balance between glutamate use for antioxidant defence and glutamate entry into anaplerotic or biosynthetic routes may influence whether glutamine metabolism is protective or profibrotic.

Evidence from other fibrotic organs further supports a link between glutamine metabolism and mitochondrial dynamics. In renal fibrosis, patients with severe disease exhibit elevated serum glutamine and increased kidney glutamine synthetase expression; experimentally, glutamine deprivation *in vitro* and *in vivo* inhibits fibroblast activation and ameliorates fibrosis. Glutamine supports mitochondrial energy generation and morphology, whereas its deprivation induces mitochondrial fission through an mTOR– mitochondrial fission process 1–Drp1 axis, indicating that modulation of glutamine metabolism can regulate mitochondrial dynamics and fibrogenesis (62). Although this evidence is not lung-specific, it provides a mechanistic framework for investigating whether similar glutamine-dependent mitochondrial remodelling occurs in IPF fibroblasts or epithelial cells.

Metabolomic evidence also supports a broader association between amino-acid metabolism, redox imbalance and fibrotic lung injury. In a polyacrylic acid-induced rat model of pulmonary fibrosis, metabolomic profiling reveals systemic changes in amino-acid, energy and redox pathways, with glutamine among the differentially regulated metabolites, linking metabolic reprogramming to oxidative stress, mitochondrial dysfunction and fibrosis (63). However, the field still lacks cell type–resolved studies that directly define how glutamine flux affects mitochondrial respiration, ROS generation and antioxidant capacity in human IPF lung tissue.

Overall, glutamine metabolism can support epithelial mitochondrial respiration and antioxidant defence, while excessive or misdirected fibroblast glutaminolysis may contribute to ROS imbalance, biosynthetic activation and fibrotic persistence. This duality reinforces the need for cell type–specific and context-aware therapeutic strategies. Future studies using human precision-cut lung slices, organoids, isotope tracing and single-cell metabolomic approaches will be required to determine whether targeting glutamine metabolism can rebalance redox and mitochondrial homeostasis without impairing epithelial repair.

### 3.4 Glutamine metabolism in immune regulation and the inflammatory microenvironment of IPF

Although IPF is not a classical inflammatory disease, persistent immune activation contributes to the profibrotic niche. Alveolar macrophages, monocyte-derived macrophages and T-cell subsets interact with fibroblasts and injured epithelial cells through cytokines, growth factors and metabolic signals. Within this microenvironment, glutamine may influence immune-cell activation by supporting TCA cycle flux, NADPH production, cytokine programmes and amino-acid-sensitive signalling pathways. However, direct evidence defining glutamine-dependent immune programmes in human IPF lungs remains limited, and many mechanistic insights are extrapolated from cancer, acute lung injury or systemic immune models.

Macrophage metabolism is altered in chronic lung diseases. Comparative analyses suggest that macrophage metabolic signatures differ between asthma, COPD and IPF, with IPF macrophages engaging both glycolytic and oxidative pathways while losing regulatory metabolites such as itaconate, thereby favouring persistent profibrotic signalling (64). Conceptual work has also highlighted an immunometabolism interface in IPF, in which PPAR*γ*, SPP1, mTOR, AMPK and HIF-1*α* integrate metabolic cues with immune activation to influence macrophage polarization, fibroblast activation and ECM production (65). In macrophages, glutamine can also sustain the IRG1/itaconate axis, which restrains NLRP3 inflammasome activation and pyroptosis; limiting glutamine reduces itaconate production, enhances NLRP3 activity and increases inflammatory cell death (66). Although these data are not all derived from IPF models, they suggest that glutamine availability may influence whether macrophages promote inflammatory persistence or resolution.

Glutamine also shapes adaptive immune responses. T cells display subset-specific dependence on glutaminase-mediated metabolism: genetic deletion or pharmacological inhibition of GLS impairs T-cell activation and Th17 differentiation while modulating Th1 and cytotoxic T-cell responses, in part through changes in mTORC1 signalling and epigenetic regulation (42). In autoimmune models, glutamine blockade reduces Th1/Th17 differentiation and modulates amino-acid transporter and mTOR activity (43). In LPSinduced acute lung injury, alanyl-glutamine treatment after injury reduces weight loss, promotes neutrophil clearance, downregulates pro-inflammatory and fibrosis-related genes and histological inflammation, while shifting the T-cell balance toward increased regulatory T cells and IL-2 and decreased Th17 cells (67). These findings suggest that glutamine availability may influence Treg/Th17 balance and immune resolution in injured lungs, although whether similar mechanisms operate in chronic IPF remains to be established.

Additional studies from non-lung settings illustrate how glutamine can reprogramme macrophage and T-cell states. CD40 ligation induces a pro-inflammatory macrophage phenotype supported by coordinated increases in fatty acid oxidation and glutamine metabolism; in this context, glutamine-to-lactate flux adjusts the NAD^+^/NADH ratio and sustains ATP-citrate lyase-dependent epigenetic activation of inflammatory genes (68). In tumour microenvironments, enhanced glutamine metabolism can promote immunosuppressive macrophage polarization, whereas tissue repair models show that macrophage-derived glutamine may support regeneration of neighbouring structural cells (69, 70). These examples should be viewed as mechanistic analogies rather than direct evidence for IPF, but they support the broader principle that glutamine metabolism can regulate immune-cell phenotype and paracrine cross-talk.

Several unresolved questions remain. It is still unclear which macrophage or T-cell subsets in human IPF lungs are most dependent on glutamine, whether glutamine-targeted interventions would preferentially dampen profibrotic immune programmes or impair host defence, and how immune and stromal cells compete for or exchange glutamine during the transition from injury to established fibrosis. Addressing these questions will be essential for designing glutamine-directed therapies that modulate the immune microenvironment without inducing clinically significant immunosuppression.

In summary, glutamine metabolism may contribute to IPF-associated immunometabolic remodelling by shaping macrophage inflammatory programmes, itaconate-linked inflammasome control and T-cell polarization. The available evidence supports immune glutamine metabolism as a plausible contributor to the profibrotic niche, but direct validation in human IPF immune subsets and lung-relevant experimental systems remains necessary.

## 4 Molecular pathways linking glutamine metabolism to fibrogenic signaling in IPF

### 4.1 mTOR, AMPK and autophagy

Glutamine acts as a critical upstream nutrient signal for mTORC1 activation in fibroblasts and other proliferating cells. In lung myofibroblasts, glutaminolysis is augmented through GLS1 upregulation, and glutamine-derived *α*-KG activates mTORC1 to enhance collagen translation. In parallel, glutamine-derived metabolites support collagen maturation by promoting proline hydroxylation and collagen stability (71). This study provides direct lung-fibrosis evidence that glutamine catabolism is coupled to both the quantity and biochemical stability of collagen protein, thereby linking nutrient availability to matrix accumulation.

Glutamine also supports anabolic and bioenergetic programmes that reinforce profibrotic growth. Parallel work in cancer-associated fibroblasts and tumour cells shows that glutamine and glutamine-derived metabolites can sustain anabolic growth and stromal–tumour metabolic crosstalk, in part through mTORlinked pathways (72, 73). Although these studies are not specific to IPF, they are consistent with a broader concept in which glutamine availability is interpreted by proliferative and matrix-producing cells as a nutrient cue that permits protein synthesis, biomass accumulation and stress adaptation.

Conversely, glutamine deprivation or blockade of glutamine uptake can suppress mTOR signalling and restore autophagy-related stress responses. In porcine intestinal epithelial cells, L-glutamine withdrawal rapidly induces autophagosome accumulation and LC3B-II expression while inactivating mTOR and MAPK/ERK pathways; glutamine repletion restores mTOR/ERK activity and cell proliferation (74). In fibrotic lungs, SLC1A5-dependent glutamine transport is highly expressed in fibrotic fibroblasts and IPF fibroblasts. Genetic or pharmacologic inhibition of SLC1A5 with V-9302 enhances fibroblast susceptibility to autophagy, suppresses mTOR, HIF and Myc signalling, impairs mitochondrial and glycolytic metabolism, and shifts fibroblast transcriptional profiles toward fibrosis-resolving states in bleomycin models (75). These findings support a model in which glutamine uptake sustains mTOR-dependent profibrotic growth, whereas limiting glutamine availability can reactivate autophagy-associated clearance programmes and partially rebalance metabolic homeostasis in IPF fibroblasts.

Other amino acids can cooperate with glutamine as nutrient cues for mTORC1, but these signals should be interpreted as complementary rather than as the central focus of glutamine-directed fibrotic metabolism. In TGF-*β*–stimulated human lung fibroblasts, arginine availability is indispensable for TGF-*β*–induced mTORC1 activation and collagen production; arginine limitation activates GCN2, suppresses mTORC1 and reduces collagen synthesis, and this deficit can be rescued by extracellular citrulline in an ASS1-dependent manner (76). Together, these data indicate that glutamine and other amino acids act as convergent nutrient signals that license mTORC1 activation, protein synthesis and matrix production in fibrotic fibroblasts, with glutamine providing a particularly important carbon and nitrogen source for collagen-producing cells.

### 4.2 TGF-*β*/Smad, Wnt/*β*-catenin, and other pro-fibrotic pathways

Glutamine metabolism is closely integrated with TGF-*β* signalling, the dominant profibrotic pathway in IPF. In lung fibroblasts, TGF-*β* induces GLS1 expression and increases glutaminolytic flux, thereby supplying metabolic intermediates required for myofibroblast activation and extracellular matrix production. Profibrotic outputs, including collagen I, PAI-1, CTGF, *α*-SMA and fibronectin, require GLS1 activity; conversely, genetic or pharmacological disruption of GLS1 attenuates these TGF-*β*–driven responses (44, 77). Mechanistically, knockdown of Smad2 or Smad3, or pharmacological inhibition of MAPK, PI3K/Akt or mTOR, abrogates TGF-*β*-induced GLS1 expression, indicating that canonical and noncanonical TGF-*β* pathways converge on glutamine metabolism (77).

This coupling is further controlled by transcriptional and epigenetic regulators. SIRT7 and FOXO4 act as endogenous negative regulators of GLS1 expression. TGF-*β* suppresses this inhibitory axis by reducing SIRT7 deacetylase activity and impairing FOXO4-mediated repression of GLS1, thereby linking TGF-*β* signalling to sustained glutamine-dependent fibrogenesis (77). Thus, rather than functioning merely as a downstream fuel source, glutamine metabolism participates in a feed-forward circuit in which TGF-*β* enhances glutaminolysis, and glutaminolysis in turn supports the bioenergetic, biosynthetic and epigenetic requirements of the activated myofibroblast phenotype.

Wnt/*β*-catenin signalling may also regulate glutamine handling in stromal cells. In pancreatic ductal adenocarcinoma, pancreatic stellate cells upregulate glutamine synthetase through a Wnt/*β*-catenin/TCF7 axis and secrete glutamine, which increases oxygen consumption and proliferation of neighbouring cancer cells. Glutamine synthetase depletion in stellate cells diminishes tumour growth, whereas *β*-catenin knockdown reduces glutamine synthetase expression and glutamine output, effects that can be rescued by glutamine synthetase overexpression (78). Although this evidence is derived from pancreatic rather than pulmonary stroma, it illustrates a principle relevant to fibrotic tissues: Wnt/*β*-catenin activation can control glutamine metabolism in stromal cells and thereby shape a pro-growth, matrix-supportive microenvironment.

Glutamine also intersects with inflammatory pathways that can modulate TGF-*β* activity indirectly. In BEAS-2B lung epithelial cells, glutamine deprivation enhances LPS-induced Akt/mTOR/IKK signalling, NF-*κ*B nuclear translocation and NF-*κ*B–dependent gene expression, whereas glutamine supplementation suppresses this cascade and improves cell viability (79). In septic rats, systemic glutamine administration after cecal ligation and puncture reduces lung NF-*κ*B activation, attenuates p38MAPK and ERK phosphorylation, increases MKP-1, diminishes pro-inflammatory cytokines and iNOS expression, prevents ARDS-like histology and improves survival (80). These observations indicate that glutamine can restrain injury-associated inflammatory signalling in some epithelial or acute injury contexts.

Importantly, these protective observations should not be interpreted as contradicting the profibrotic role of glutaminolysis in activated fibroblasts. Instead, they underscore the cell type– and context-dependent nature of glutamine metabolism. In epithelial cells or acute inflammatory injury, adequate glutamine may support barrier integrity, antioxidant defence and stress resolution, whereas in activated fibroblasts persistent glutamine catabolism can support collagen synthesis, apoptosis resistance and matrix accumulation. This distinction is critical for therapeutic translation: glutamine-targeted interventions in IPF may need to suppress pathological fibroblast-directed glutaminolysis while preserving epithelial repair and immune competence.

### 4.3 Epigenetic regulation driven by glutamine-derived metabolites

Glutaminolysis generates *α*-KG and other TCA cycle intermediates that serve as cofactors for dioxygenases such as TET DNA demethylases and JmjC histone demethylases, thereby linking glutamine metabolism to epigenetic regulation of fibrotic gene programmes. Reviews of fibrotic diseases outside the lung, including chronic pancreatitis and cardiac remodelling, emphasise that metabolic reprogramming of fibroblasts and immune cells—shifts in glycolysis, lipid metabolism and amino-acid utilization—supports ECM overproduction and that targeting amino-acid pathways, including glutamine, may offer antifibrotic benefit (81). In macrophage immunometabolism, accumulation of TCA intermediates such as succinate and citrate, as well as itaconate, exerts non-metabolic signalling functions, while alternatively activated macrophages require *α*-KG–dependent epigenetic reprogramming to establish anti-inflammatory gene expression (82, 83). Emerging work on histone and non-histone lactylation further illustrates how metabolic by-products of glycolysis can drive epigenetic programmes that promote disease progression and treatment resistance through activation of oncogenic and immunosuppressive transcriptional networks (84).

**Figure 3:**
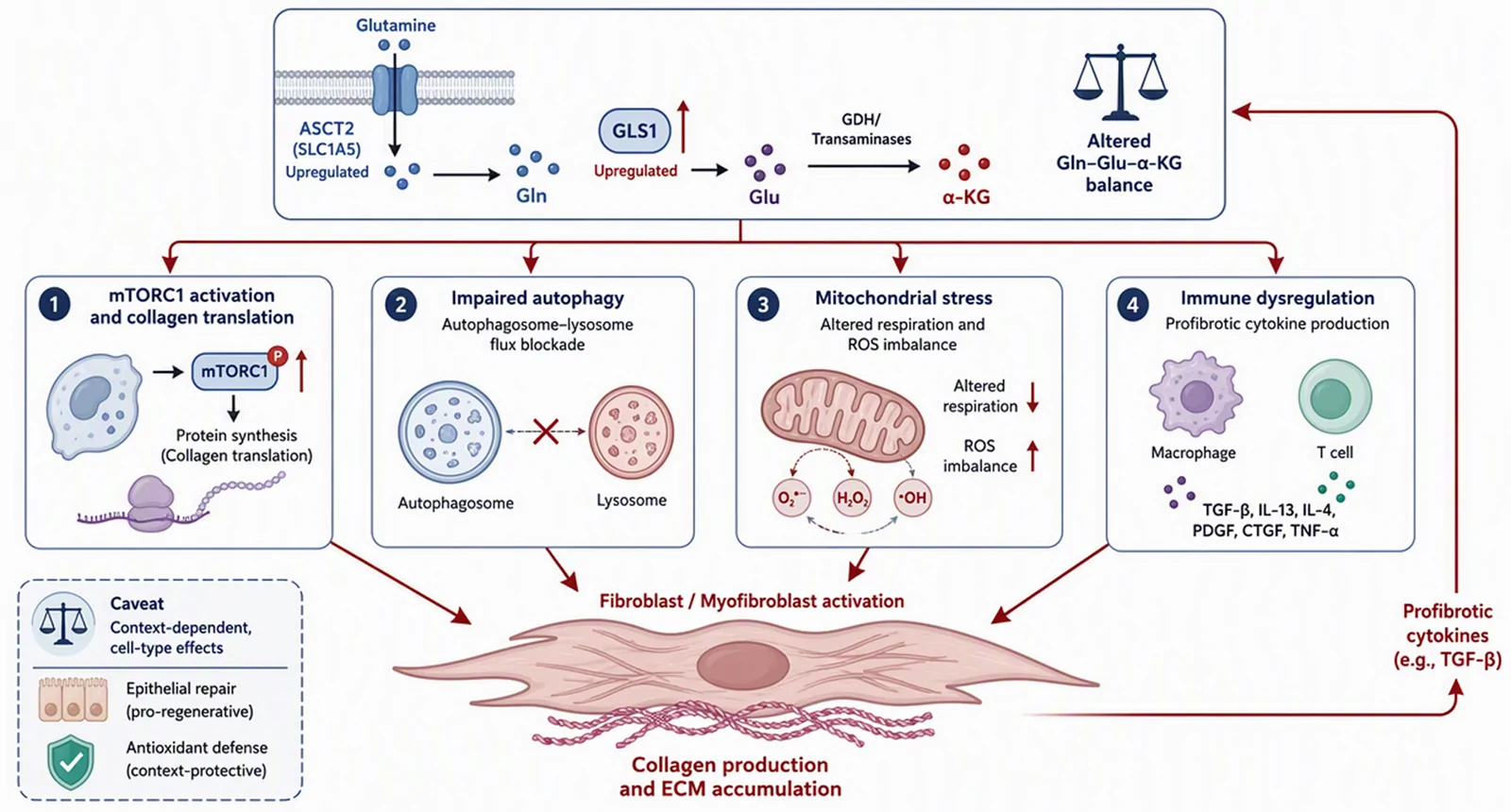
Integrated network linking dysregulated glutamine metabolism to pulmonary fibrosis. Dysregulated glutamine metabolism, characterised by increased glutamine uptake, GLS1 upregulation and an altered glutamine–glutamate–*α*-ketoglutarate balance, contributes to aberrant mTORC1 activation, impaired autophagy, mitochondrial stress with altered respiration and ROS imbalance, and profibrotic immune responses. These interconnected pathways converge on fibroblast/myofibroblast activation, collagen biosynthesis and excessive extracellular matrix deposition. The scheme is conceptual and highlights context-dependent effects across cell types: excessive fibroblast-directed glutaminolysis may promote matrix accumulation, whereas epithelial glutamine utilisation may support repair, barrier integrity and antioxidant defence.

Within the lung, epithelial cells and fibroblasts in pulmonary fibrosis display metabolic and epigenetic alterations that contribute to EMT, chronic oxidative stress and pathological remodelling (85, 86). IPF fibroblasts show enhanced glutaminolysis and resistance to apoptosis; inhibition of glutaminolysis reduces expression of anti-apoptotic proteins XIAP and survivin, increases H3K27me3 levels and decreases activity of the H3K27 demethylase JMJD3 (87). Chromatin immunoprecipitation confirms that JMJD3 directly binds the XIAP and survivin promoters in a glutamine-dependent manner, and exogenous *α*-KG partially restores JMJD3 function and XIAP expression under glutamine-deficient conditions (87). These findings provide direct evidence that glutamine-derived *α*-KG supports an epigenetic state favouring apoptosis resistance in IPF fibroblasts and, together with broader immunometabolic data, support the concept of a metabolism–epigenetic axis in which glutamine metabolism stabilises profibrotic gene expression programmes. These molecular observations can be integrated into a unified network model in which dysregulated glutamine metabolism coordinates mTOR signalling, autophagy impairment, mitochondrial stress, redox imbalance and immune dysregulation to drive progressive fibrosis (**Figure 3**). Because direct evidence linking glutamine metabolism to irreversible mitochondrial membrane-potential loss in IPF remains limited, the model emphasises mitochondrial stress, altered respiration and ROS imbalance rather than a single fixed mitochondrial endpoint.

## 5 Evidence from animal models and human studies on glutamine metabolism in IPF

Experimental animal models and ex vivo studies provide strong proof-of-concept that glutamine metabolism is required for fibroblast activation and collagen production in pulmonary fibrosis. Lung myofibroblasts are recognised as the primary executors of pulmonary fibrosis, and recent advances in characterising their pathobiology, spanning cell-of-origin, lineage heterogeneity and metabolic regulation, have positioned cellular metabolism as a key determinant of their pathogenic phenotype and a promising therapeutic target (88); consistent with this framework, fibroblast GLS1 is upregulated in bleomycin-induced lung fibrosis and pharmacologic GLS1 inhibition with CB-839 attenuates both bleomycin- and TGF-*β*1-induced pulmonary fibrosis, supporting GLS1 as a viable antifibrotic target *in vivo* (17). Similarly, blocking glutamine uptake via ASCT2 knockdown in bleomycin-treated mice reduces pulmonary glutamate, *α*-KG, GSH and ATP levels, mitigates histological injury and collagen deposition, lowers hydroxyproline and serum fibrosis markers, and decreases expression of *α*-SMA, collagen I*α*1, TGF-*β* receptor II and inflammatory cytokines, indicating that glutamine transport is essential for maintaining the profibrotic metabolic state (89). Modulation of glutathione and cysteine handling also influences bleomycin responses: *γ*-glutamyl transpeptidase-deficient mice exhibit an altered early inflammatory profile and develop markedly less fibrosis and collagen accumulation, implicating thiol and amino-acid metabolism in the fibrotic outcome (90). More broadly, TGF-*β*1 drives a conserved metabolic reprogramming in activated fibroblasts across fibrotic organs, rewiring cellular energetics toward glycolysis and enhanced glutamine utilisation to sustain the high bioenergetic and biosynthetic demands of the myofibroblastic phenotype, with redirected acetylCoA providing an additional link between metabolic state and regulatory protein acetylation (91); in the lung, this glutamine-dependent programme is orchestrated through SMAD2/3, PI3K, mTORC2 and PDGFR signalling with SIRT7 and FOXO4 as endogenous suppressors of GLS1 expression that are attenuated by TGF-*β*, and *in vivo* GLS1 inhibition with CB-839 reduces bleomycin-induced fibrosis (77). Other agents with metabolic effects, such as peimine, also ameliorate bleomycin-induced pulmonary fibrosis by reducing collagen deposition, EMT, inflammation and oxidative stress while modulating alanine, aspartate, glutamate and D-glutamine/D-glutamate metabolism in lung tissue (92). Beyond the lung, glutaminase inhibition reduces fibroblast proliferation and collagen I/III and *α*-SMA expression in airway scar fibroblasts from iatrogenic laryngotracheal stenosis, suggesting that targeting glutaminolysis may have broader anti-fibrotic potential in airway scarring (93). Reviews on collagen-targeted therapies emphasise that enzymes involved in collagen amino-acid supply, including glycine and proline biosynthetic pathways fed by glutamine, are emerging drug targets in lung fibrosis (94), and preclinical toxicology of glutaminase antagonists such as DON indicates minimal dermal fibroblast toxicity at analgesic doses, supporting further development of glutamine-targeted interventions (95).

Human lung tissue and fibroblast studies corroborate these findings. TGF-*β*–dependent myofibroblast differentiation requires glutamine conversion to glutamate by GLS, and downstream metabolism of glutamate into *α*-KG via dehydrogenase or transaminases is dispensable for collagen synthesis; instead, glutamate is channelled via PSAT1 and ALDH18A1/P5CS into *de novo* glycine and proline, which are required for collagen production (96). IPF fibroblasts display enhanced glutaminolysis and rely on glutamine for collagen production and survival *in vitro* (88, 91, 96). At the systemic level, two sample Mendelian randomization using GWAS data provides genetic evidence that lower circulating glutamine levels are causally associated with increased IPF risk (inverse correlation; odds ratio ≈0.75), supporting a role for glutamine availability in disease susceptibility (97). Although direct plasma or BALF glutamine/glutamate profiling in IPF is limited, studies in collagen vascular disease–associated ILD and acute-onset diffuse ILD demonstrate that plasma amino-acid signatures are altered and can be integrated with markers such as KL-6 to improve ILD diagnosis (98). Similarly, multivariate models based on plasma amino-acid profiles have been used as screening indices for non-small-cell lung cancer (99, 100), illustrating that systemic amino-acid patterns carry diagnostic information in lung pathology.

From a translational perspective, these animal and human data have complementary strengths and weaknesses. Animal models provide clear causal links between genetic or pharmacologic manipulation of glutamine pathways and fibrotic outcomes, but they are typically based on single acute injuries in young animals without comorbidities, and interventions are often applied at very early time points. Human studies, in contrast, capture the complexity of long standing disease but are largely cross sectional, rely on limited tissue availability and are confounded by factors such as age, malnutrition, renal function and concomitant medications that also influence amino acid profiles (6, 7, 98–100). Bridging these gaps will require longitudinal studies that integrate intrapulmonary glutamine pathway measurements, systemic metabolomics and clinical phenotyping, as well as models that better recapitulate the chronic and heterogeneous nature of IPF.

## 6 Therapeutic potential of targeting glutamine metabolism in IPF

### 6.1 Glutamine transport, glutaminase inhibitors and downstream metabolic modulators

Direct targeting of glutamine metabolism can be conceptualised at three levels: inhibition of glutamine uptake, blockade of glutaminolysis and modulation of downstream metabolic routing. Among transporters, SLC1A5/ASCT2 has received the greatest attention in pulmonary fibrosis. SLC1A5 is highly expressed in fibrotic lung fibroblasts and IPF fibroblasts, and genetic or pharmacological blockade of SLC1A5 reduces glutamine uptake, suppresses mTOR, HIF and Myc signalling, perturbs mitochondrial and glycolytic metabolism, and shifts fibroblast transcriptional profiles toward less fibrogenic states in bleomycin-induced pulmonary fibrosis (75). Consistently, ASCT2 inhibition mitigates bleomycin-induced lung injury and collagen deposition, reduces pulmonary glutamate, *α*-KG, GSH and ATP levels, and lowers expression of *α*-SMA, collagen I*α*1, TGF-*β* receptor II and inflammatory cytokines (89). These observations support glutamine transport blockade as a plausible antifibrotic strategy.

However, transporter targeting will require careful attention to cell-type specificity. Glutamine uptake may support epithelial repair, antioxidant defence and immune-cell fitness in some contexts, so systemic inhibition of transporters such as SLC1A5 could have undesirable effects on barrier integrity or host defence. Other amino-acid transport systems may also be relevant. SLC7A5/LAT1 functions primarily as a sodium-independent exchanger for large neutral amino acids and can use intracellular glutamine to support leucine uptake, thereby indirectly linking glutamine availability to mTORC1 activation. SLC38A2/SNAT2 can mediate glutamine and alanine transport; in pulmonary fibrosis, TGF-*β* coordinates SLC38A2-mediated alanine import with GPT2-dependent alanine synthesis in normal and IPF lung fibroblasts, sustaining glycolytic flux, TCA cycle intermediates, glutamate and proline biosynthesis required for myofibroblast differentiation (101). Evidence from tumour immunology further suggests that SLC38A2-dependent glutamine acquisition can influence dendritic-cell function and T-cell priming, indicating that immune consequences should be monitored when this transporter system is targeted systemically (102).

Downstream of transport, GLS1 remains the most direct and pharmacologically tractable node in the glutaminolytic pathway. In IPF-relevant models, fibroblast GLS1 is induced by TGF-*β* and contributes to collagen production, myofibroblast differentiation and fibrotic remodelling (17, 77, 96). Pharmacological GLS1 inhibition with CB-839 attenuates experimental pulmonary fibrosis and reduces profibrotic fibroblast outputs, supporting GLS1 as a candidate antifibrotic target (17). Outside the lung, GLS inhibition also suppresses myofibroblast formation in cardiac fibroblasts, where glutaminolysis supplies *α*-KG for histone demethylation and bioenergetic remodelling (103), and reduces fibroblast proliferation and matrix-gene expression in iatrogenic laryngotracheal scar fibroblasts (93). These studies strengthen the concept that glutaminolysis is a conserved metabolic vulnerability of activated fibroblasts.

Oncology studies provide an important pharmacological foundation for GLS inhibition but should be interpreted mainly as evidence of druggability, safety experience and metabolic feasibility rather than as disease-specific evidence for IPF. CB-839 and other GLS inhibitors have been evaluated in multiple cancer contexts, including haematological malignancies, hepatocellular carcinoma and lung tumours, where they reduce glutamine-dependent metabolic fitness, clonogenic survival or treatment resistance (104–108). These data support the feasibility of pharmacological GLS targeting, but the therapeutic window in chronic fibrotic lung disease may differ substantially from oncology because patients with IPF are often older, have impaired lung function and receive long-term background antifibrotic therapy.

For clinical translation, several issues need to be resolved before glutamine-directed therapies can be advanced in IPF. First, only a subset of patients may exhibit a strongly glutamine-dependent fibrotic endotype, so biomarker-based enrichment may be required. Candidate biomarkers include GLS1 or SLC1A5 expression, glutamine-pathway gene signatures, collagen-synthesis signatures, imaging markers of active fibrosis and plasma or BALF amino-acid profiles. Second, long-term inhibition of glutamine metabolism could affect epithelial repair, immune surveillance, intestinal homeostasis and systemic nitrogen balance. Third, metabolic plasticity may allow fibroblasts to bypass single-node inhibition through alternative anaplerotic or amino-acid pathways. Therefore, early-phase studies should prioritise pharmacodynamic confirmation of on-target pathway modulation, careful safety monitoring and rational combination strategies with established antifibrotics.

### 6.2 Indirect modulation via mTOR/AMPK/autophagy and mitochondrial protection

Indirect targeting of glutamine-linked pathways via AMPK activation and mTOR inhibition has shown robust antifibrotic activity in preclinical lung models. Metformin, a canonical AMPK activator, attenuates silica-induced pulmonary fibrosis in several rodent models: it lowers TGF-*β*1, TNF-*α* and IL-1*β*, suppresses EMT markers such as *α*-SMA and vimentin while restoring E-cadherin, and enhances autophagy in association with increased p-AMPK and reduced p-mTOR (109). Importantly, metformin has also been shown to accelerate the resolution of established lung fibrosis in human IPF myofibroblasts and in a bleomycin model through AMPK-dependent myofibroblast deactivation and apoptosis (110). Additional work shows that metformin remodels endothelial-to-mesenchymal transition, protects vascular morphology, reverses SiO_2_-induced toxicity and oxidative stress, and dampens epithelial, macrophage and fibroblast activation via AMPK-dependent autophagy (111, 112). Other agents such as tracheloside and bryodulcosigenin also act through AMPK: tracheloside activates AMPK, suppresses NOX4 and oxidative stress, and inhibits TGF-*β*– or stiffness-induced myofibroblast differentiation (113), whereas bryodulcosigenin activates AMPK, downregulates TGF-*β*1/Smad2/3 signalling, inhibits EMT and oxidative stress, and loses its benefits when co-treated with the AMPK inhibitor compound C (114). A broad review supports AMPK as a central integrator of energy stress with anti-inflammatory, antioxidant and antifibrotic responses in the lung (115). On the mTOR side, dysregulated PI3K/AKT/mTOR signalling contributes to abnormal fibroblast growth, survival and impaired autophagy in pulmonary fibrosis, and mTOR is considered an attractive target in IPF and radiation-induced lung fibrosis (116). In bleomycin-induced fibrosis, rapamycin reduces fibrotic burden and EMT, preserves E-cadherin and limits fibronectin in alveolar epithelial cells; in TGF-*β*1–treated epithelial cells, mTOR inhibition attenuates EMT, reduces S6K and Smad2/3 phosphorylation and reverses cytoskeletal changes (117). Because glutamine is a key nutrient cue for mTORC1 and supports mitochondrial and redox metabolism, AMPK activators and mTOR inhibitors are likely to indirectly modulate glutamine usage and its downstream consequences without directly blocking GLS or transporters. Clinically, the challenge will be to balance antifibrotic efficacy with systemic metabolic effects, identify IPF subgroups with pronounced AMPK/mTOR dysregulation, and determine how these agents synergise or interfere with glutamine-directed therapies.

### 6.3 Combination strategies and clinical translation challenges

A further caveat is that glutamine metabolism is not uniformly pathogenic across all lung cell types or disease stages. Several injury models indicate that glutamine supplementation can preserve epithelial viability, suppress NF-*κ*B activation, reduce oxidative stress or attenuate acute inflammatory injury (79, 80, 118). These protective effects are likely to be most relevant in epithelial, endothelial or immune compartments during acute stress, where glutamine supports antioxidant defence, nucleotide synthesis, barrier repair and immune-cell function. In contrast, persistent fibroblast-directed glutaminolysis in the fibrotic niche may promote collagen biosynthesis, apoptosis resistance and extracellular matrix accumulation. Therefore, therapeutic strategies should avoid indiscriminate suppression of glutamine metabolism and instead aim for cell type–aware, disease stage–specific modulation of pathological glutamine flux.

Experience with combination antifibrotic therapy in IPF provides a template for integrating metabolic interventions. Real-world cohorts suggest that pirfenidone plus nintedanib is generally tolerable, with diarrhoea and anorexia as the main adverse events; some data indicate a slower FVC decline after initiating combination therapy, although matched analyses show similar FVC trajectories to monotherapy but higher rates of non-serious gastrointestinal toxicity (119). In advanced silicosis, both drugs improve lung function and fibrosis in mice, and low-dose combination therapy outperforms monotherapy; multi-omics analyses implicate immune pathways and metabolic processes in lipids, nucleotides and carbohydrates as shared mechanisms, underscoring the feasibility of multi-pathway interference in fibrotic lung disease (120). Framed by the “fibrometabolism” concept, IPF increasingly appears as a metabolic disease of myofibroblasts, epithelium and immune cells, making glutaminolysis, glycolysis and fatty-acid oxidation logical partners for combination with established antifibrotics (121).

Emerging phase II and III trials in IPF, such as those testing BI 1015550 on top of standard antifibrotics, highlight the expectation that new therapies will often be add-on agents and will be evaluated using FVC decline and composite exacerbation/hospitalisation/death endpoints (122, 123). At the same time, qualitative work shows that patients with IPF want to participate in trials but often find information unclear and processes exclusionary, and call for more inclusive design and tailored communication (124). For glutamine-targeted strategies, key challenges will be to develop metabolic or transcriptomic biomarkers to identify “glutamine-dependent” IPF subgroups, anticipate metabolic plasticity and bypass pathways that may blunt single-node inhibition, integrate glutamine-directed agents safely with pirfenidone, nintedanib and new drugs, and embed meaningful patient engagement into eligibility criteria, endpoints and trial conduct.

In practical terms, an incremental research agenda could start with small mechanistic studies that add glutamine targeted agents to background antifibrotic therapy in biomarker selected patients, with intensive monitoring of safety, lung function, imaging and immune and metabolic readouts. If early signals are favourable, larger randomised trials could then test whether such combinations meaningfully alter trajectories of FVC decline, exacerbation rates and survival compared with standard of care alone. Throughout this process, transparent communication of potential risks and benefits, including uncertainties about long term metabolic effects, will be crucial to maintain patient trust and ensure that trial designs align with patient priorities.

## 7 Conclusions and perspectives

Emerging data position dysregulated glutamine metabolism as a central, cross-cutting contributor to IPF rather than a secondary bystander. Across structural and immune compartments, glutamine fuels myofibroblast activation and collagen biosynthesis, supports redox and mitochondrial adaptation under stress, and shapes macrophage and T-cell phenotypes in the fibrotic niche. Glutaminolysis intersects with mTOR/AMPK, TGF-*β*/Smad and Wnt/*β*-catenin signalling, as well as *α*-KG-dependent epigenetic enzymes, thereby stabilising profibrotic, apoptosis-resistant programmes. Preclinical and ex vivo data show that perturbing glutamine uptake, catabolism or downstream routing can attenuate experimental lung fibrosis and reprogramme fibroblasts toward a less pathogenic state. These features make glutamine metabolism an attractive therapeutic axis that is selectively upregulated in IPF effector cells and is pharmacologically tractable through GLS inhibition, amino-acid transporter blockade, downstream metabolic enzymes and nutrient-sensing modulators.

At the same time, glutamine biology is highly context-dependent. Epithelial and immune cells may require glutamine for antioxidant defence, barrier repair, host defence and resolution of acute inflammatory injury, whereas activated fibroblasts may exploit persistent glutaminolysis to sustain matrix production and apoptosis resistance. Thus, glutamine-targeted therapy should not be viewed as simple blanket inhibition. Instead, future approaches will need to define when, where and in which cell populations glutamine metabolism is pathological. Cell type–resolved profiling, spatial metabolomics, organoid and precision-cut lung-slice systems, and biomarker-driven clinical studies will be essential to identify glutamine-dependent IPF endotypes and to distinguish protective from profibrotic glutamine flux.

Metabolic plasticity and redundant anaplerotic circuits may blunt single-target strategies and demand rational combinations. Integration of glutamine-directed agents with pirfenidone, nintedanib, nerandomilast and emerging therapies will require careful safety monitoring, pharmacodynamic confirmation of target engagement and patient-centred trial design. Particular attention should be paid to epithelial repair, immune competence, gut toxicity, systemic nitrogen balance and long-term metabolic adaptation. If these challenges can be addressed, targeting pathological glutamine metabolism has the potential not only to slow functional decline but also to reshape the trajectory of IPF as a chronic, more controllable disease.
